# Targeted energy transfer and modal energy redistribution in automotive drivetrains

**DOI:** 10.1007/s11071-016-3034-4

**Published:** 2016-09-02

**Authors:** E. Motato, A. Haris, S. Theodossiades, M. Mohammadpour, H. Rahnejat, P. Kelly, A. F. Vakakis, D. M. McFarland, L. A. Bergman

**Affiliations:** 1 Wolfson School of Mechanical, Electrical and Manufacturing Engineering, Loughborough University, Loughborough, LE11 3TU UK; 2 Ford Werke GmbH, Cologne, Germany; 3 Department of Mechanical Science and Engineering, University of Illinois at Urbana-Champaign, Urbana, IL 61801 USA; 4 Department of Aerospace Engineering, University of Illinois at Urbana-Champaign, Urbana, IL 61801 USA

**Keywords:** Targeted energy transfer, Nonlinear energy sink, Automotive drivetrain, Modal energy redistribution

## Abstract

The new generations of compact high output power-to-weight ratio internal combustion engines generate broadband torsional oscillations, transmitted to lightly damped drivetrain systems. A novel approach to mitigate these untoward vibrations can be the use of nonlinear absorbers. These act as Nonlinear Energy Sinks (NESs). The NES is coupled to the primary (drivetrain) structure, inducing passive irreversible targeted energy transfer (TET) from the drivetrain system to the NES. During this process, the vibration energy is directed from the lower-frequency modes of the structure to the higher ones. Thereafter, vibrations can be either dissipated through structural damping or consumed by the NES. This paper uses a lumped parameter model of an automotive driveline to simulate the effect of TET and the assumed modal energy redistribution. Significant redistribution of vibratory energy is observed through TET. Furthermore, the integrated optimization process highlights the most effective configuration and parametric evaluation for use of NES.

## Introduction

Recent developments have resulted in downsized turbo-charged engines replacing the large capacity naturally aspirated variety [1]. Higher output power-to-weight ratio is the trend in modern powertrain engineering [2–4]. Furthermore, turbo-charged, direct injection engines achieve better fuel efficiency and reduced emissions (e.g. the new generation 1.0 l turbocharged Ford EcoBoost three-cylinder engine produces the same power as that of an older naturally aspirated 1.6 l four-cylinder engine). However, there is an inherent tendency for increased torsional oscillations, thus exacerbated Noise, Vibration and Harshness (NVH) because of larger variations in combustion torque. There are a plethora of engine-induced NVH phenomena such as body boom [5], clutch whoop [6], transmission rattle [7–9], axle whine [10, 11] and driveline clonk [12, 13] which are resolved during the Development process. Much of these phenomena are induced by engine order vibration [2] or impulsive action leading to elasto-acoustic modal response [14]. There have been various palliative measures, such as clutch torsional dampers [15], Dual Mass Flywheel (DMF) [16] and DMFs with centrifugal pendulum vibration absorbers in order to reduce automotive drivetrain vibrations [17]. Most of these remedial actions are quite expensive or add to the powertrain inertia, which is contrary to the prevailing light weight and compact philosophy. Furthermore, most palliative measures are tuned to operate in a narrow frequency band, usually targeting the dominant engine order frequency.

Nonlinear vibration absorbers (NESs) are light weight devices, possessing essentially nonlinear stiffness and low damping [18–21]. When connected to a linear (primary) system, the NES nonlinearity couples with the vibration modes of the primary system, allowing irreversible energy transfer between them [22]. Thus, the excess energy of the primary system is irreversibly scattered from low-frequency modes to high-frequency ones, which is then dissipated through structural damping. Some of the excess energy is retained within the NES itself [23]. This action leads to a reduction in the vibration amplitudes of the primary system in a broadband manner, which is due to the strong nonlinearity of the NES [24–26]. The energy dissipation rate of the primary structure can be controlled by adjusting the damping content of the NES [27].

Numerical and experimental investigations of systems exhibiting translational motions have already shown that an NES can induce energy redistribution between assumed modes, resulting in targeted energy transfers from low-to-high frequencies. In the available literature [28–34], the mechanism of this targeted energy transfer (TET) or “energy pumping” is well documented for structures coupled to strongly nonlinear mechanical oscillators. The effects of NES on the dissipation and redistribution of energy were studied by Quinn et al. [22] in a two-DoF linear structure, subjected to impulsive excitation. It has been shown that the energy redistribution is directly related to changes in the overall damping capabilities of the system. Sapsis et al. [35] defined two appropriate measures to quantify these effects due to variation in damping and stiffness during the operation of the primary linear system coupled to a NES. Three different types of NES were introduced, and their effects on the instantaneous and average damping were analysed. Wierschem et al. [36] complemented the work reported in [35] by providing experimental evidence for enhanced damping produced by the NES in a two-DoF structure. It was demonstrated that the proper selection of the absorber’s stiffness and inertia allowed it to efficiently attenuate vibration energy over a broad frequency band. In a study with increased complexity Al-Shudeifat et al. [27] used two vibro-impact NESs to induce energy redistribution between the modes of a nine-story building structure. Numerical evidence showed that in regions where NESs are active, the high-frequency modes of the structure are net recipients of energy, implying that the vibration energy is efficiently dissipated at these modes through structural damping.

This paper presents numerical evidence of energy redistribution between the torsional structural modes of an automotive drivetrain. The normalized effective damping factor [35] is utilized to identify the structural modes which absorb or release energy. This is an initial study to determine the factors which maximize the transferred energy from the primary (drivetrain) structure to the NES, whilst dissipating the energy through structural damping. The main aim is to establish whether the TET mechanism can be conceptually used in automotive drivetrains. This approach has not hitherto been reported in the literature in automotive powertrain applications.

## Drivetrain model

**Fig. 1:**
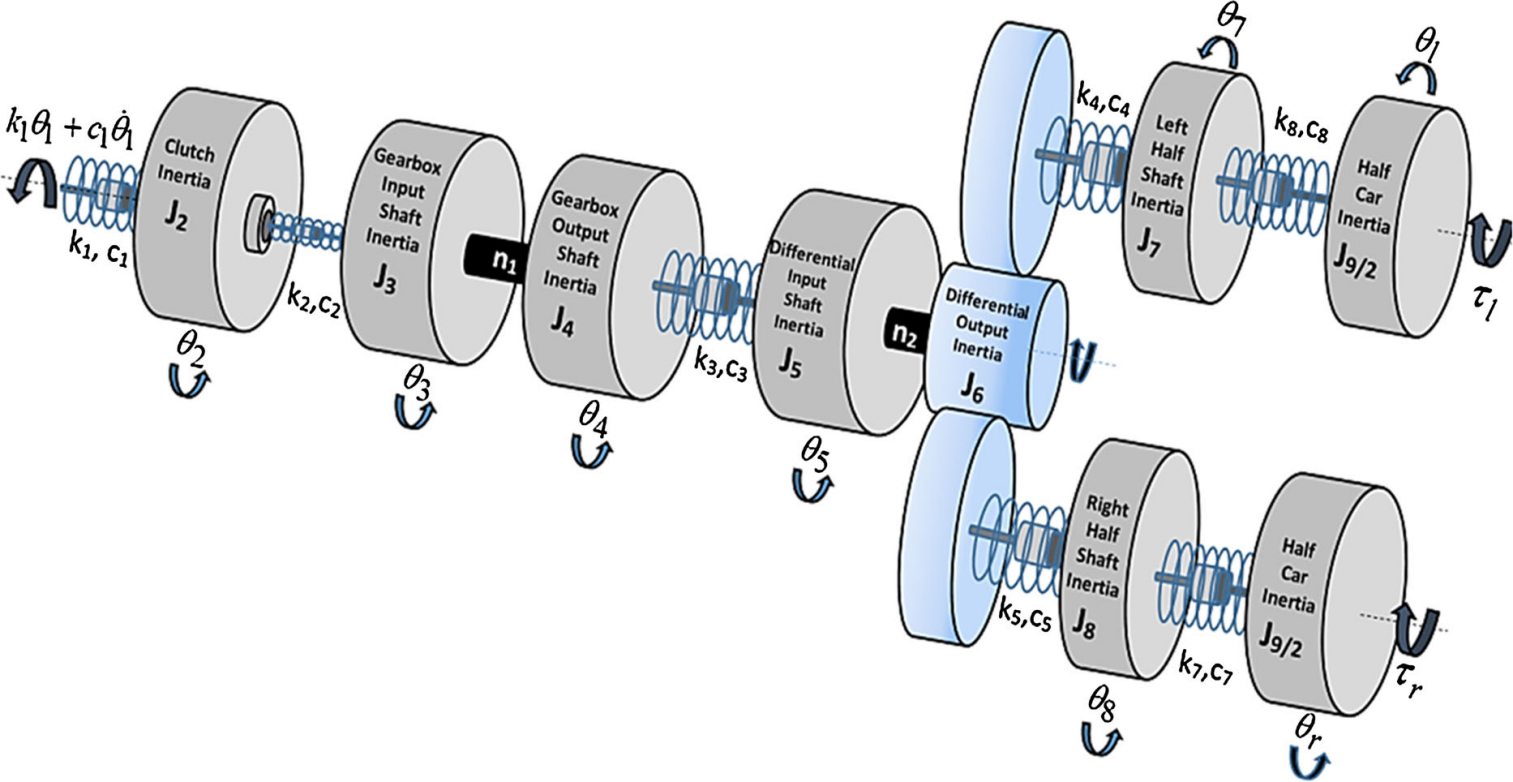
The drivetrain model

A front wheel drive (FWD) vehicle with a 3-cylinder engine is studied. The drivetrain is equipped with a clutch torsional damper and a solid mass flywheel. The powertrain comprises the engine, flywheel, clutch, gearbox, differential and transaxle half-shafts (Fig. 1). The engine-flywheel assembly (inertia $J_{1}$) is not included in the model in order to reduce the total DoF. The drivetrain is powered by the transferred engine torque variation as an input to the system model.

The main torsional DOF are those of the clutch friction disc $\theta _2$, the gearbox input/output shaft $\theta _3$/$\theta _4$, the differential input/output shaft $\theta _5$/$\theta _6$, the left axle half-shaft $\theta _7$, the right axle half-shaft $\theta _8$, the left tyre $\theta _{l}$ and the right tyre $\theta _{r}$. The constant ratio $n_{1}$ of the engaged gear pair couples the angular position of the gearbox input shaft ($\theta _{3}$) to that of the gearbox output shaft ($\theta _{4}$) as a constraint. Similarly, the constant ratio $n_{2}$ couples the angular position of the differential input shaft ($\theta _{5}$) to its output shaft ($\theta _{6}$). The model parameters are the inertia of the clutch assembly ($J_{2}$), the gearbox input shaft ($J_{3}$), the gearbox output shaft ($J_{4}$), the differential input shaft ($J_{5}$), the differential output shaft ($J_{6}$), the right ($J_{7}$) and the left ($J_{8}$) axle half-shafts and the vehicle inertia ($J_{9}$). The torsional stiffness coefficients $k_1 ,\;k_2 ,\;k_3 ,\;k_4 ,\;k_5 ,\;k_6$, $k_7$ and $k_8$ represent the connection restraints between the aforementioned driveline components (Fig. 1).

The input torque at the flywheel end is given by $k_1 \theta _1 +c_1 \dot{\theta }_1$, where $\theta _1$ is the flywheel rotation, $k_{1}$ is the coupling stiffness of flywheel to the clutch disc and $c_{1}$ is its corresponding structural damping coefficient. The rolling resistance torques from the left and right tyres are $\tau _l$ and $\tau _r$, respectively, and are defined as [37]:

1 $$\begin{aligned} \tau _r =\tau _l =\frac{1}{2}\left( {F_\mathrm{D} \cdot r_w +T_\mathrm{R} } \right) \end{aligned}$$

where $$F_\mathrm{D} $$ is the total aerodynamic drag force, $$r_w $$ is the laden rolling radius of the tyre and $$T_\mathrm{R} $$ is the resisting torque generated at the contact patch. The aerodynamic drag resistance force is defined as:

2 $$\begin{aligned} F_D =\frac{1}{2}\rho \cdot V^{2}\cdot C_\mathrm{D} \cdot A_\mathrm{f} \end{aligned}$$

where $\rho$ is the density of air, *V* is the vehicle forward speed, $C_\mathrm{D}$ is the dimensionless coefficient of drag and $A_{\mathrm{f}}$ is the frontal area of the vehicle. The rolling resistance torque is:

3 $$\begin{aligned} T_R =4\left\{ {\left( P \right) ^{\alpha }\left( N \right) ^{\beta }\left( {A+B\cdot V+C\cdot V^{2}} \right) } \right\} r_w \end{aligned}$$

where $P = 250$ kPa is the tyre pressure, $N = 3374.64$ (*N*) is the normal load (a quarter of vehicle’s weight), $\alpha = -0.003$ is the tyre pressure coefficient, $\beta = 0.97$ is the normal force coefficient and the constants $A = 84e-4\,\hbox {m}^{2}$, $B = 6.2-4$ m-s and $C = 1.6e-4\,\hbox {s}^{2}$. Typical values for the vehicle component inertias, transmission ratios and the stiffness parameters were provided by the industrial partners. The equations of motion take the matrix form:

4 $$\begin{aligned} \mathbf{J}\left[ {{\begin{array}{c} {\ddot{\theta }_2 } \\ {\ddot{\theta }_4 } \\ {\ddot{\theta }_6 } \\ {\ddot{\theta }_7 } \\ {\ddot{\theta }_8 } \\ {\ddot{\theta }_l } \\ {\ddot{\theta }_r } \\ \end{array} }} \right] +\mathbf{C}\left[ {{\begin{array}{c} {\dot{\theta }_2 } \\ {\dot{\theta }_4 } \\ {\dot{\theta }_6 } \\ {\dot{\theta }_7 } \\ {\dot{\theta }_8 } \\ {\dot{\theta }_l } \\ {\dot{\theta }_r } \\ \end{array} }} \right] +\mathbf{K}\left[ {{\begin{array}{c} {\theta _2 } \\ {\theta _4 } \\ {\theta _6 } \\ {\theta _7 } \\ {\theta _8 } \\ {\theta _l } \\ {\theta _r } \\ \end{array} }} \right] =\left[ {{\begin{array}{c} {k_1 \theta _1 +c_1 \dot{\theta }_1 } \\ 0 \\ 0 \\ 0 \\ 0 \\ {\tau _l } \\ {\tau _r } \\ \end{array} }} \right] \quad \end{aligned}$$

where the inertial matrix is:

5 $$\begin{aligned} \mathbf{J}=\left[ {{\begin{array}{ccccccc} {J_2 }&{} 0&{} 0&{} 0&{} 0&{} 0&{} 0 \\ 0&{} {\left( {J_3 {\varvec{\upeta }}_\mathbf{1} ^{2}+J_4 } \right) }&{} 0&{} 0&{} 0&{} 0&{} 0 \\ 0&{} 0&{} {\left( {J_5 {\varvec{\upeta }}_\mathbf{2} ^{2}+J_6 } \right) }&{} 0&{} 0&{} 0&{} 0 \\ 0&{} 0&{} 0&{} {J_7 }&{} 0&{} 0&{} 0 \\ 0&{} 0&{} 0&{} 0&{} {J_8 }&{} 0&{} 0 \\ 0&{} 0&{} 0&{} 0&{} 0&{} {J_l }&{} 0 \\ 0&{} 0&{} 0&{} 0&{} 0&{} 0&{} {J_r } \\ \end{array} }} \right] \end{aligned}$$

And the stiffness matrix is:

6 $$\begin{aligned} \mathbf{K}=\left[ {{\begin{array}{ccccccc} {k_1 +k_2 }&{} {-k_2 {\varvec{\upeta }}_\mathbf{1} }&{} 0&{} 0&{} 0&{} 0&{} 0 \\ {-k_2 {\varvec{\upeta }}_\mathbf{1} }&{} {\left( {k_2 {\varvec{\upeta }}_\mathbf{1} ^{2}+k_3 } \right) }&{} {-k_3 {\varvec{\upeta }}_\mathbf{2} }&{} 0&{} 0&{} 0&{} 0 \\ 0&{} {-k_3 {\varvec{\upeta }}_\mathbf{2} }&{} {\left( {k_3 {\varvec{\upeta }}_\mathbf{2} ^{2}+k_4 +k_5 } \right) }&{} {-k_4 }&{} {-k_5 }&{} 0&{} 0 \\ 0&{} 0&{} {-k_4 }&{} {\left( {k_4 +k_6 } \right) }&{} 0&{} {-k_6 }&{} 0 \\ 0&{} 0&{} {-k_5 }&{} 0&{} {\left( {k_5 +k_7 } \right) }&{} 0&{} {-k_7 } \\ 0&{} 0&{} 0&{} {-k_6 }&{} 0&{} {k_6 }&{} 0 \\ 0&{} 0&{} 0&{} 0&{} {-k_7 }&{} 0&{} {k_7 } \\ \end{array} }} \right] \end{aligned}$$

The structural damping matrix $\mathbf{C}$ is calculated using the Caughey method [38] as:

7 $$\begin{aligned} \mathbf{C}=\mathbf{J}\times \mathbf{V}\times \mathbf{Z}\times \mathbf{V}^{T}\times \mathbf{J} \end{aligned}$$

where **V** is the mass normalized modal matrix obtained through the generalized eigenvalue problem, **J** is the inertia matrix and $\mathbf{Z}$ is a diagonal matrix containing the modal damping ratios in the form:

8 $$\begin{aligned} \mathbf{Z}=\left[ {{\begin{array}{ccccccc} {2\zeta _1 \omega _1 }&{} 0&{} 0&{} 0&{} 0&{} 0&{} 0 \\ 0&{} {2\zeta _2 \omega _2 }&{} 0&{} 0&{} 0&{} 0&{} 0 \\ 0&{} 0&{} {2\zeta _3 \omega _3 }&{} 0&{} 0&{} 0&{} 0 \\ 0&{} 0&{} 0&{} {2\zeta _4 \omega _4 }&{} 0&{} 0&{} 0 \\ 0&{} 0&{} 0&{} 0&{} {2\zeta _5 \omega _5 }&{} 0&{} 0 \\ 0&{} 0&{} 0&{} 0&{} 0&{} {2\zeta _6 \omega _6 }&{} 0 \\ 0&{} 0&{} 0&{} 0&{} 0&{} 0&{} {2\zeta _7 \omega _7 } \\ \end{array} }} \right] \nonumber \\ \end{aligned}$$

with $\zeta _i$ as the *i*th damping ratio, corresponding to the natural frequency.

Matlab/Simulink is used to integrate the equations of motion (4). Flywheel angular displacement data were measured from a vehicle equipped with a similar drivetrain. For the operating conditions presented in here, the vehicle is driven on a race track with a sweep speed range with a steady 25 % throttle. Experimental measurements of the vehicle acceleration (longitudinal and lateral), throttle position, engine mean torque and speed were acquired through a Controller Area Network (CAN) protocol and stored. Two additional sensors were also used to directly measure the angular velocities of the flywheel and the gearbox input shaft at variable sampling rate. The corresponding angular displacements and accelerations were obtained through signal integration and differentiation.

The modal damping ratios in matrix Eq. (8) are tuned so that the numerical simulations match the measurements both qualitatively and quantitatively (in both spectral and temporal domains). Thus, a variety of damping ratio combinations is obtained with the objective of matching the responses of the gearbox input shaft. Table 1 lists the selected numerical damping ratios, the approximate natural frequencies and the corresponding mode shapes:

**Table 1:** Damping ratios, approximate natural frequencies and the corresponding mode shapes

Damping ratio	Natural frequency (Hz)	Main contributing DoF
$\zeta _1 =0.0065$	$\omega _1 =1\;$	$\theta _2 ,\theta _l$
$\zeta _2 =0.0065$	$\omega _2 =2$	$\theta _8 ,\theta _r$
$\zeta _3 =0.055$	$\omega _3 =17$	$\theta _2 ,\theta _7$
$\zeta _4 =0.065$	$\omega _4 =18$	$\theta _8 ,\theta _2$
$\zeta _5 =0.75$	$\omega _5 =60$	$\theta _2 ,\theta _4$
$\zeta _6 =0.65$	$\omega _6 =700$	$\theta _2 ,\theta _4$
$\zeta _7 =0.75$	$\omega _8 =1100$	$\theta _2 ,\theta _6$

**Fig. 2:**
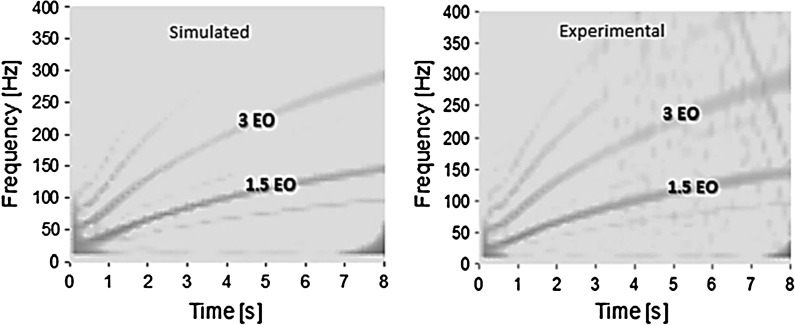
CWT for experimental and simulated gearbox input velocity in first gear with 25 % open throttle

**Fig. 3:**
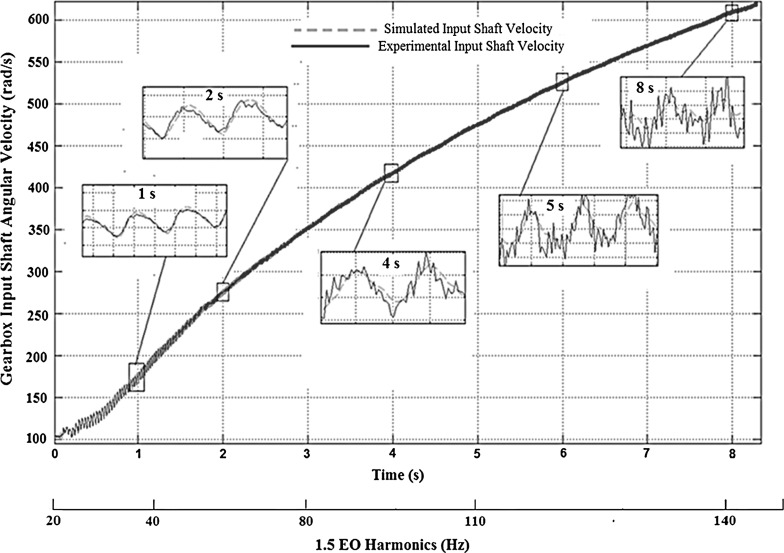
Comparison of the gearbox input shaft velocity for numerical and experimental time histories (corresponding to 1.5 EO Harmonic contributions)

**Fig. 4:**
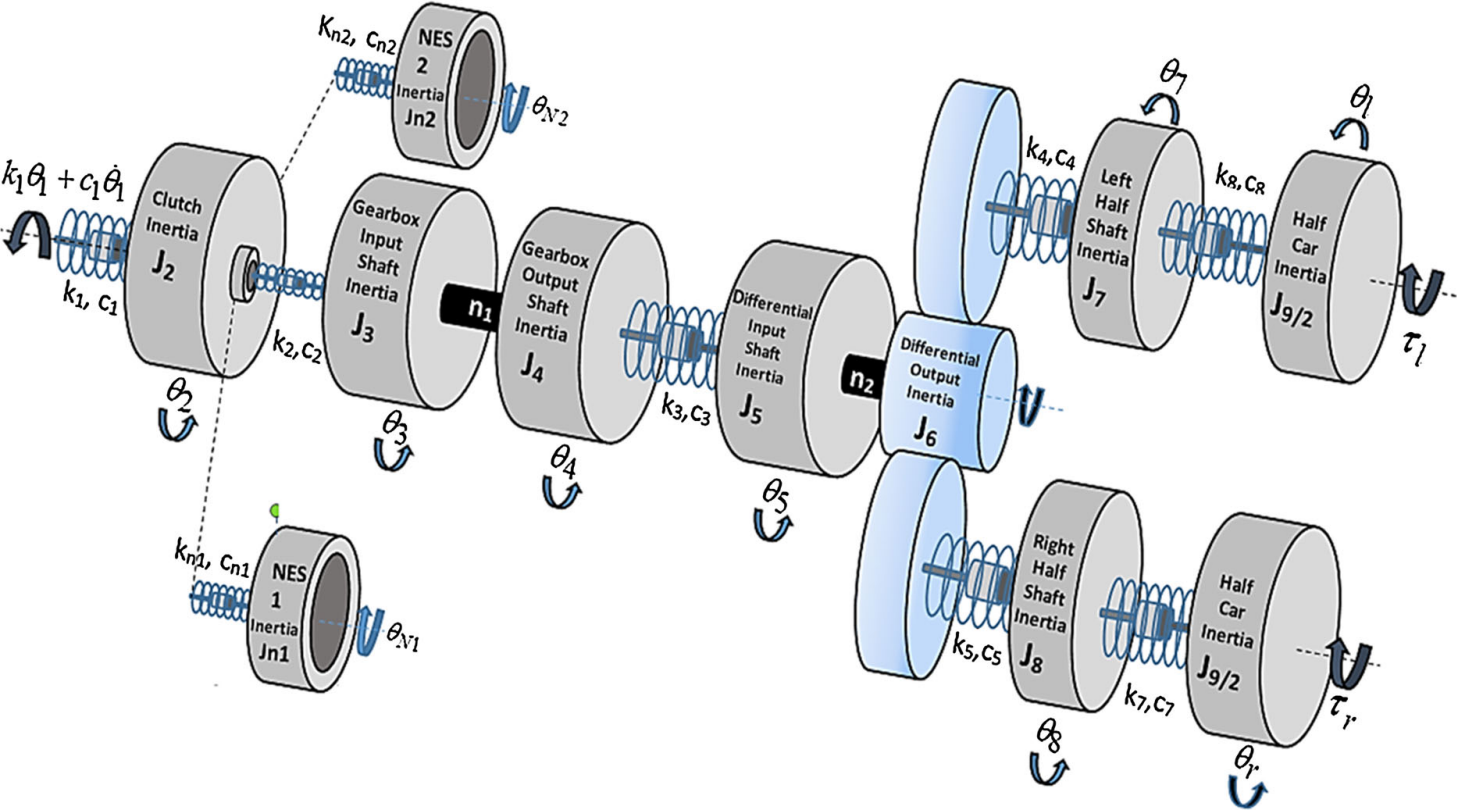
The drivetrain model with two nonlinear absorbers in parallel with the clutch disc

The model validation in the frequency domain is carried out using the continuous wavelet transform (CWT). This is because of the transient nature of the examined powertrain manoeuvres. The CWT analysis shows the fundamental engine order (EO) response to be at 1.5 multiple of crankshaft rotational frequency, which would be expected for a 3-cylinder 4-stroke engine (combustion occurs thrice over two crankshaft revolutions) [2]. The experimental and numerical predicted CWT spectra of the gearbox input shaft velocity for the drivetrain accelerating in first gear with 25 % open throttle are shown in Fig. 2. The predictions conform well to the experimental results. The analysis is extended to different manoeuvres in higher gears, showing the same degree of conformance. Figure 3 shows the prediction-measurement comparison in the time domain, where the insets to the figure are for different instants in the manoeuvre, exhibiting the predictions (in discontinuous line) and the experimental measurements (continuous line) for the gearbox input shaft velocity with the drivetrain operating in the first gear. The scale of the corresponding engine harmonics is provided below the time axis.

## Drivetrain model with two coupled nonlinear vibration absorbers

The drivetrain model represented by Eq. (4) is modified to accommodate two cubic NESs connected in parallel to the clutch inertia (Fig. 4).

The differential equations of motion become:

9 $$\begin{aligned}&\mathbf{J}_N \left[ {{\begin{array}{c} {\ddot{\theta }_2 } \\ {\ddot{\theta }_4 } \\ {\ddot{\theta }_6 } \\ {\ddot{\theta }_7 } \\ {\ddot{\theta }_8 } \\ {\ddot{\theta }_l } \\ {\ddot{\theta }_r } \\ {\ddot{\theta }_{n1} } \\ {\ddot{\theta }_{n2} } \\ \end{array} }} \right] +\mathbf{C}_N \left[ {{\begin{array}{c} {\dot{\theta }_2 } \\ {\dot{\theta }_4 } \\ {\dot{\theta }_6 } \\ {\dot{\theta }_7 } \\ {\dot{\theta }_8 } \\ {\dot{\theta }_l } \\ {\dot{\theta }_r } \\ {\dot{\theta }_{n1} } \\ {\dot{\theta }_{n2} } \\ \end{array} }} \right] +\mathbf{K}_N \left[ {{\begin{array}{c} {\theta _2 } \\ {\theta _4 } \\ {\theta _6 } \\ {\theta _7 } \\ {\theta _8 } \\ {\theta _l } \\ {\theta _r } \\ {\theta _{n1} } \\ {\theta _{n2} } \\ \end{array} }} \right] \nonumber \\&\qquad +\left[ {{\begin{array}{c} {k_{n2} \left( {\theta _2 -\theta _{n2} } \right) ^{3}+k_{n1} \left( {\theta _2 -\theta _{n1} } \right) ^{3}} \\ 0 \\ 0 \\ 0 \\ 0 \\ 0 \\ 0 \\ {k_{n1} \left( {\theta _{n1} -\theta _2 } \right) ^{3}} \\ {k_{n2} \left( {\theta _{n2} -\theta _2 } \right) ^{3}} \\ \end{array} }} \right] \nonumber \\&\quad =\left[ {{\begin{array}{c} {k_1 \theta _1 +c_1 \dot{\theta }_1 } \\ 0 \\ 0 \\ 0 \\ 0 \\ {\tau _l } \\ {\tau _r } \\ 0 \\ 0 \\ \end{array} }} \right] \end{aligned}$$

The new inertia and stiffness matrices are:

10 $$\begin{aligned} \mathbf{J}_N= & {} \left[ {{\begin{array}{ccccccccc} {J_2 }&{} 0&{} 0&{} 0&{} 0&{} 0&{} 0&{} 0&{} 0 \\ 0&{} {\left( {J_3 {\varvec{\upeta }}_\mathbf{1} ^{2}+J_4 } \right) }&{} 0&{} 0&{} 0&{} 0&{} 0&{} 0&{} 0 \\ 0&{} 0&{} {\left( {J_5 {\varvec{\upeta }}_\mathbf{2} ^{2}+J_6 } \right) }&{} 0&{} 0&{} 0&{} 0&{} 0&{} 0 \\ 0&{} 0&{} 0&{} {J_7 }&{} 0&{} 0&{} 0&{} 0&{} 0 \\ 0&{} 0&{} 0&{} 0&{} {J_8 }&{} 0&{} 0&{} 0&{} 0 \\ 0&{} 0&{} 0&{} 0&{} 0&{} {J_l }&{} 0&{} 0&{} 0 \\ 0&{} 0&{} 0&{} 0&{} 0&{} 0&{} {J_r }&{} 0&{} 0 \\ 0&{} 0&{} 0&{} 0&{} 0&{} 0&{} 0&{} {J_{n1} }&{} 0 \\ 0&{} 0&{} 0&{} 0&{} 0&{} 0&{} 0&{} 0&{} {J_{n2} } \\ \end{array} }} \right] \nonumber \\ \end{aligned}$$

11 $$\begin{aligned} \mathbf{K}_N= & {} \left[ {{\begin{array}{ccccccccc} {\left( {k_1 +k_2 } \right) }&{} {-k_2 {\varvec{\upeta }}_\mathbf{1} }&{} 0&{} 0&{} 0&{} 0&{} 0&{} 0&{} 0 \\ {-k_2 {\varvec{\upeta }}_\mathbf{1} }&{} {\left( {k_2 {\varvec{\upeta }}_\mathbf{1} ^{2}+k_3 } \right) }&{} {-k_3 {\varvec{\upeta }}_\mathbf{2} }&{} 0&{} 0&{} 0&{} 0&{} 0&{} 0 \\ 0&{} {-k_3 {\varvec{\upeta }}_\mathbf{2} }&{} {\left( {k_3 {\varvec{\upeta }}_\mathbf{2} ^{2}+k_4 +k_5 } \right) }&{} {-k_4 }&{} {-k_5 }&{} 0&{} 0&{} 0&{} 0 \\ 0&{} 0&{} {-k_4 }&{} {\left( {k_4 +k_6 } \right) }&{} 0&{} {-k_6 }&{} 0&{} 0&{} 0 \\ 0&{} 0&{} {-k_5 }&{} 0&{} {\left( {k_5 +k_7 } \right) }&{} 0&{} {-k_7 }&{} 0&{} 0 \\ 0&{} 0&{} 0&{} {-k_6 }&{} 0&{} {k_6 }&{} 0&{} 0&{} 0 \\ 0&{} 0&{} 0&{} 0&{} {-k_7 }&{} 0&{} {k_7 }&{} 0&{} 0 \\ 0&{} 0&{} 0&{} 0&{} 0&{} 0&{} 0&{} 0&{} 0 \\ 0&{} 0&{} 0&{} 0&{} 0&{} 0&{} 0&{} 0&{} 0 \\ \end{array} }} \right] \end{aligned}$$

The drivetrain model in Eq. (9) is hereinafter referred to as the *Active NES model*. This designation should not be confused with the purely passive nature of the NES; rather, the characterization as “active” is meant to denote an operational NES, inducing strongly nonlinear effects under transient dynamic conditions. This point is emphasized due to the nonlinear (nonlinearizable) nature of the stiffness of the NES. Therefore, it can interact with arbitrary modes of the drivetrain, passively absorbing and redistributing vibration energy over a broad range of frequencies. It follows that although *local*, the NES can induce *global* effects in system dynamics to which it is attached (in this case the drivetrain system) [18].

**Fig. 5:**
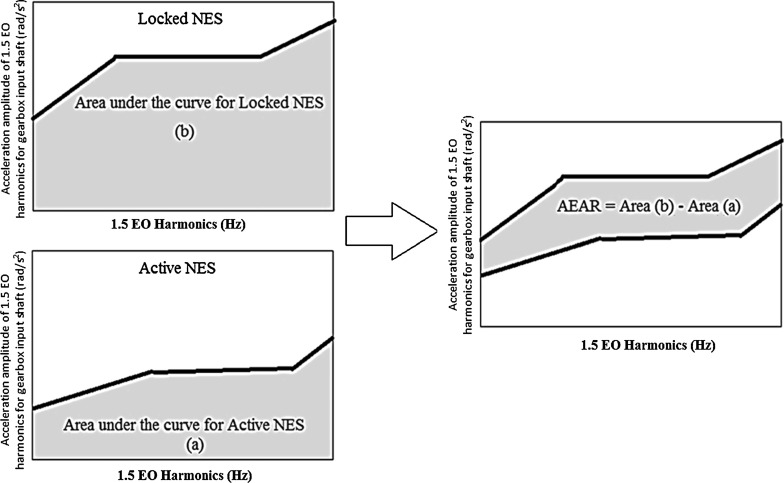
Area of effective acceleration reduction (AEAR)

The parameters $$\theta _{n1} $$, $$k_{n1} $$, $$J_{n1} $$ and $$c_{n1} $$ are the angular position, stiffness, inertia and damping coefficients associated with the first NES. In a similar manner, the parameters $$\theta _{n2} $$, $$k_{n2} ,J_{n2} $$ and $$c_{n2} $$ correspond to the second NES. The $$\left( {9\times 9} \right) $$ linear damping matrix of the *Active NES model* is given by:

12 $$\begin{aligned} \mathbf{C}_N =\left[ {{\begin{array}{cc} {\mathbf{C}_x }&{} {\mathbf{c}_a^T } \\ {\mathbf{c}_a }&{} {\mathbf{c}_d } \\ \end{array} }} \right] \end{aligned}$$

where the matrices $\mathbf{c}_a$ and $c_d$ are defined as:

13 $$\begin{aligned} \mathbf{c}_a= & {} \left[ {{\begin{array}{ccccccc} {-c_{n1} }&{} 0&{} 0&{} 0&{} 0&{} 0&{} 0 \\ {-c_{n2} }&{} 0&{} 0&{} 0&{} 0&{} 0&{} 0 \\ \end{array} }} \right] \end{aligned}$$

14 $$\begin{aligned} \mathbf{c}_d= & {} \left[ {{\begin{array}{cc} {c_{n1} }&{} 0 \\ 0&{} {c_{n2} } \\ \end{array} }} \right] \end{aligned}$$

The matrix $\mathbf{C}_x$ is based on the operation of a drivetrain with *locked* (non-active) NESs, since the performance of the absorbers are evaluated by comparing the response of the *Active NES model* to that of a model with the two NES inertias simply added to the clutch in the inertia matrix $\mathbf{J}$ (*locked* model). The equations of motion describing the *locked NES model* take the following form:

15 $$\begin{aligned} {\hat{\mathbf{J}}}\left[ {{\begin{array}{c} {\ddot{\theta }_2 } \\ {\ddot{\theta }_4 } \\ {\ddot{\theta }_6 } \\ {\ddot{\theta }_7 } \\ {\ddot{\theta }_8 } \\ {\ddot{\theta }_l } \\ {\ddot{\theta }_r } \\ \end{array} }} \right] +\mathbf{C}_x \left[ {{\begin{array}{c} {\dot{\theta }_2 } \\ {\dot{\theta }_4 } \\ {\dot{\theta }_6 } \\ {\dot{\theta }_7 } \\ {\dot{\theta }_8 } \\ {\dot{\theta }_l } \\ {\dot{\theta }_r } \\ \end{array} }} \right] +\mathbf{K}\left[ {{\begin{array}{c} {\theta _2 } \\ {\theta _4 } \\ {\theta _6 } \\ {\theta _7 } \\ {\theta _8 } \\ {\theta _l } \\ {\theta _r } \\ \end{array} }} \right] =\left[ {{\begin{array}{c} {k_1 \theta _1 +c_1 \theta _1 } \\ 0 \\ 0 \\ 0 \\ 0 \\ {\tau _l } \\ {\tau _r } \\ \end{array} }} \right] \end{aligned}$$

with the inertia matrix given as:

16 $$\begin{aligned} {\hat{\mathbf{J}}}=\left[ {{\begin{array}{ccccccc} {J_2 +J_{n1} +J_{n2} }&{} 0&{} 0&{} 0&{} 0&{} 0&{} 0 \\ 0&{} {\left( {J_3 {\varvec{\upeta }}_\mathbf{1} ^{2}+J_4 } \right) }&{} 0&{} 0&{} 0&{} 0&{} 0 \\ 0&{} 0&{} {\left( {J_5 {\varvec{\upeta }}_\mathbf{2} ^{2}+J_6 } \right) }&{} 0&{} 0&{} 0&{} 0 \\ 0&{} 0&{} 0&{} {J_7 }&{} 0&{} 0&{} 0 \\ 0&{} 0&{} 0&{} 0&{} {J_8 }&{} 0&{} 0 \\ 0&{} 0&{} 0&{} 0&{} 0&{} {J_l }&{} 0 \\ 0&{} 0&{} 0&{} 0&{} 0&{} 0&{} {J_r } \\ \end{array} }} \right] \nonumber \\ \end{aligned}$$

and the damping matrix defined as:

17 $$\begin{aligned} \mathbf{C}_x ={\hat{\mathbf{J}}}\times {\hat{\mathbf{V}}}\times \mathbf{Z}\times {\hat{\mathbf{V}}}^{T}\times {\hat{\mathbf{J}}} \end{aligned}$$

where ${\hat{\mathbf{V}}}$ is the mass normalized modal matrix of the locked system, obtained through the generalized eigenvalue problem for ${\hat{\mathbf{J}}}$ and $\mathbf{K}$.

The performance of the two parallel NESs is assessed with respect to the reduction in acceleration amplitude of the 1.5 EO harmonic at the location of the gearbox input shaft. This frequency harmonic carries most of the vibration energy of the drivetrain, and, therefore it is an acceptable benchmark for assessing the induced oscillations in the drivetrain [39]. By comparing the amplitude of gearbox input shaft acceleration for the 1.5 EO harmonics when (*a*) the drivetrain is operating with the two parallel NESs active against (*b*) the drivetrain operating with the inertias of the two parallel NES simply added to the clutch inertia (*locked NESs*), the regions of effective operation for each NES can be identified (Fig. 5). Subtracting the area under the curve defined in (*a*) from the area under the curve defined in (*b*), the area of effective acceleration reduction (AEAR) is obtained (Fig. 5).

The higher the value of AEAR, the better the performance of the NES is in reducing the system torsional vibrations. For both cases (i.e. the *Locked* and the *Active NES*), the acceleration amplitude corresponding to the 1.5 EO harmonic is calculated using:

18 $$\begin{aligned} A_i =\sqrt{P_i \times f_{i} } \end{aligned}$$

where $$f_{i} $$ is the frequency and $$A_i $$ is the acceleration amplitude of the 1.5 EO harmonic of the gearbox input shaft and $$P_i $$ is its corresponding power spectral density. The power spectral density is computed using the Matlab command *pwelch*, which uses Welch’s method [40].

**Fig. 6:**
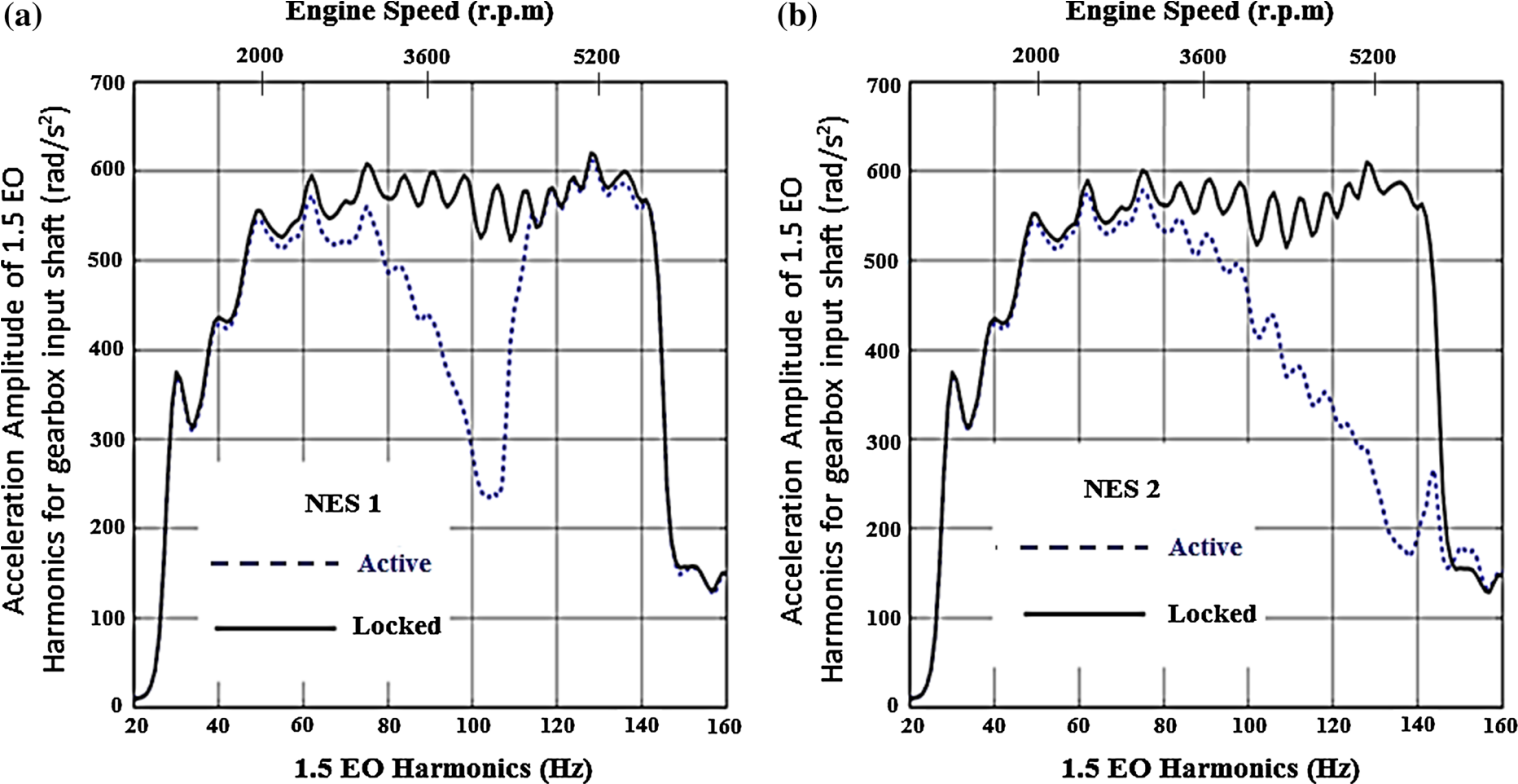
Spectra of the 1.5 EO harmonic acceleration amplitude of the transmission input shaft for **a** NES1 and **b** NES2 in 1st gear with 25 % open throttle

A large number of simulations are carried out in Matlab/Simulink for a range of NES stiffness, damping and inertia combinations with the objective of maximizing AEAR. In total, 140,000 parameter combinations are examined for a typical vehicle manoeuvre which lasts 8 s, corresponding to the accelerative motion of the vehicle in first gear with 25 % open throttle. During this manoeuvre, the speed of the engine increases from (approximately) 1000 to 6000 rpm. The data obtained from these simulations are introduced in the optimization software $\hbox {CAMEO}^\circledR$ (of AVL), which uses a neural network/genetic algorithm [41] subject to pre-defined parameter constraints in order to determine the NES parameter combination which would provide the highest AEAR. Initially, CAMEO generates a multi-layer perceptron neural network which describes the input/output map of the data provided. For this particular case, the NES parameters are used as inputs and the corresponding AEAR is assumed as the output (objective function). The valid neural network model obtained is used by CAMEO in conjunction with external parameter constraints (for stiffness, damping and inertia) to generate a hyper-dimensional surface. Finally, a genetic algorithm locates the point on the surface that maximizes AEAR. The total inertia for the two NESs added is constrained to be smaller than 15 % of the gearbox input shaft inertia, thus representing a modest and acceptable addition to the drivetrain overall inertia and mass. The optimized NES parameters, which lead to the maximum reduction in the 1.5 EO acceleration (at the input shaft), are presented in Table 2.

**Table 2:** Nonlinear absorber parameters

NES 1	NES 2
$k_{n1} =3.9e4\,\hbox {N m/rad}^{3}$	$k_{n2} =9.5e5\,\hbox {N m/rad}^{3}$
$J_{n1} =6$ % of the input shaft inertia	$J_{n2} =9$ % of the input shaft inertia
$c_{n1} =0.001$ N m s/rad	$c_{n2} =0.001$ N m s/rad

The frequency range where a significant reduction in the acceleration amplitude of the 1.5 EO input shaft harmonic occurs can be divided into two regions. The first region is covered by the NES with the lower stiffness (NES1) between 60–110 Hz, whilst the second region is covered by the NES with the higher stiffness (NES2) between 80 and 140 Hz. These regions are identified by simulating the drivetrain model equipped first only with NES1 (Fig. 6a) and then only with NES2 (Fig. 6b).

**Fig. 7:**
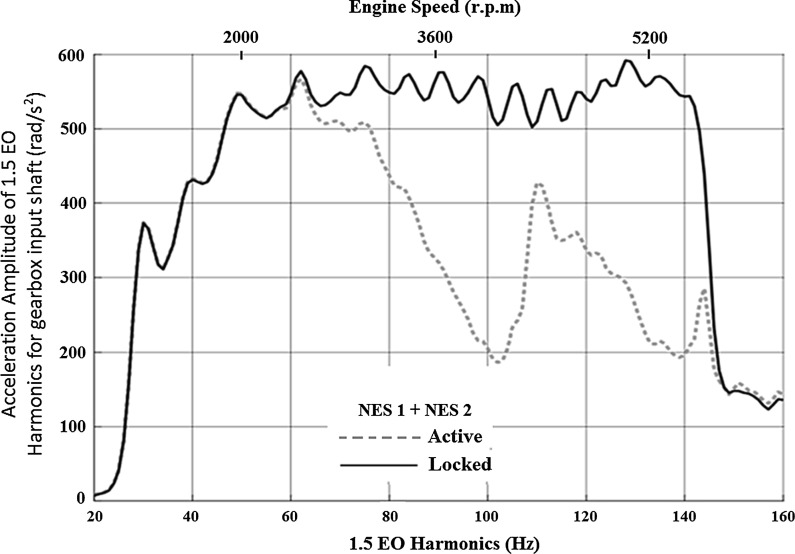
Spectrum of the 1.5 EO harmonic acceleration amplitude of the transmission input shaft for both NESs acting in first gear with 25 % open throttle

The corresponding spectrum of the 1.5 EO acceleration amplitude of the input shaft for both the NESs acting simultaneously is shown in Fig. 7. It can be seen that the use of two NESs, coupled to the drivetrain, increases the frequency range effectiveness in a synergistic manner.

**Fig. 8:**
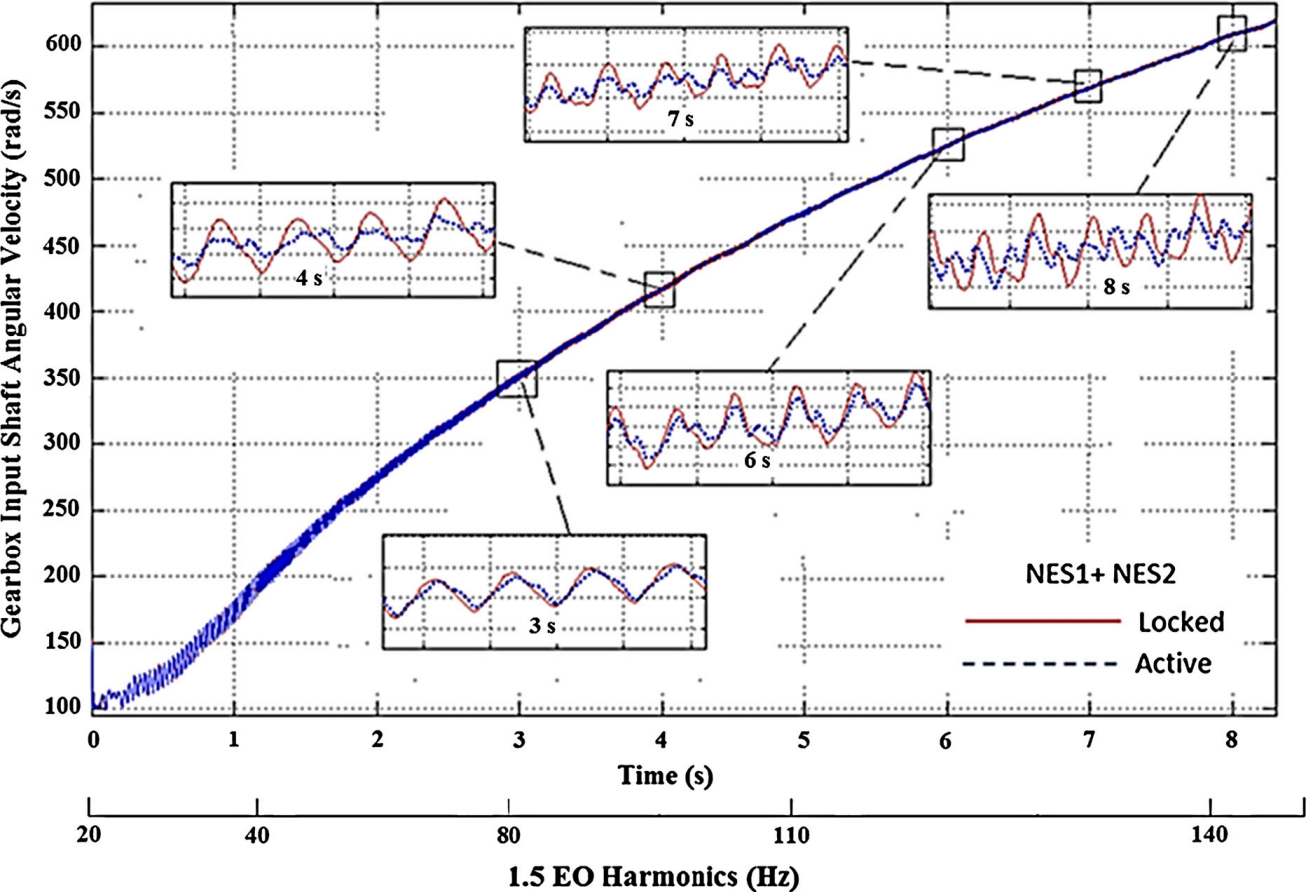
Transmission input shaft velocity time history for the drivetrain model with *active* and *locked* NES operating in first gear with 25 % open throttle

The time history of the gearbox input shaft angular velocity for the optimized system with two NESs (data presented in Table 2) is shown in Fig. 8. The amplitude of oscillations is reduced within a significant part of the manoeuvre, starting approximately at 3 s. This specific timeframe corresponds to the frequency range, where the influence of the 1.5 EO is diminished (approximately at 60–140 Hz). As the inset to the figure shows, around 4 s into the manoeuvre the angular velocity fluctuations of the gearbox input shaft (due to the 1.5 EO harmonic) for the active system is substantially reduced, when compared to the *locked* system. This reduction corresponds to the minimum value of the gearbox input shaft acceleration amplitude (up to 200 rad/s$^{2}$), which occurs around 100 Hz (the 1.5 EO harmonic, Fig. 7). At around 6 s, the amplitude of oscillations of the *active* system increases again, similar to that of the *locked* system. This behaviour occurs around 110 Hz (Fig. 7), where the gearbox input shaft acceleration increases to 420 rad/s$^{2}$. The other time domain insets (at 7 and 8 s) show that the reduction in oscillations is effective for the rest of the manoeuvre.

**Fig. 9:**
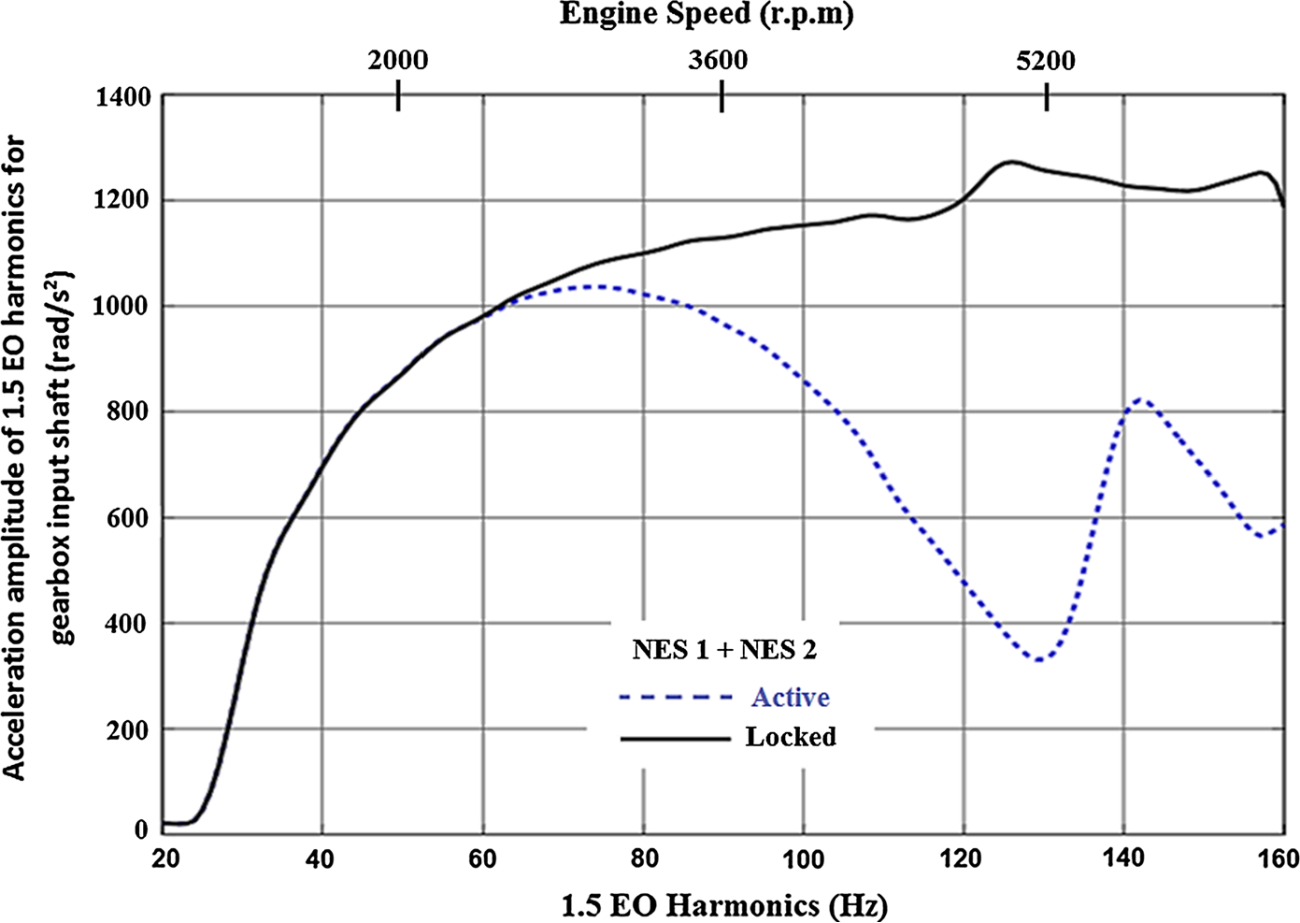
Spectrum of the acceleration amplitude of 1.5 EO harmonics for the transmission input shaft for both active NESs in third gear with full throttle

The effectiveness of the absorbers in suppressing vibrations in different manoeuvres is also examined. Figure 9 shows the spectrum of the acceleration amplitude of the transmission for 1.5 EO harmonic in a manoeuvre, lasting 34 s in third gear with full throttle. It can be seen that in this case the two NESs also reduce the amplitude of oscillations over a broad range of frequencies (70–160 Hz).

**Fig. 10:**
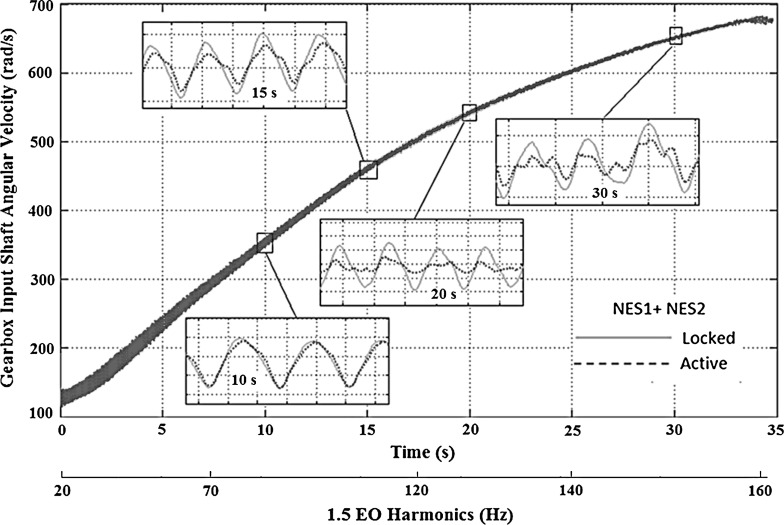
Transmission input shaft velocity time history for the drivetrain model with *active* and *locked NES* operating in third gear with full throttle

The time history of the transmission input shaft’s angular velocity for the optimized system operating in third gear with full throttle is shown in Fig. 10. The amplitude of oscillations is reduced within a significant part of the manoeuvre, commencing approximately at 15 s. This timeframe corresponds to the frequency range, where the influence of 1.5 EO is diminished (70–160 Hz, as shown in Fig. 9). In the corresponding inset to the figure at 20 s the angular velocity fluctuations of the gearbox input shaft for the *active* system are reduced substantially, when compared with those of the *locked* system. This reduction continues to the minimum value of the gearbox input shaft acceleration at 1.5 EO harmonic (approximately 350 rad/s$^{2}$), which occurs at around 130 Hz (Fig. 9). The other time domain insets (at 20 and 30 s) show that the reduction in oscillations induced by the NES is effective throughout the rest of the manoeuvre.

## Modal energy redistribution, effective damping and energy dissipation

It has already been shown that the essential nonlinear stiffness of the two NESs induces significant changes in the dynamics of the primary structure (drivetrain), reducing the torsional vibrations over a broad band of frequencies. This may occur in two possible ways. Firstly, the NES operates in a 1:1 resonance with the primary system. This resonance can occur in isolation, involving a single frequency or in cascade resonant captures, where the response jumps between different frequencies. This phenomenon generates one-directional energy transfer from the highly energetic structural modes to the NES, where the energy is trapped and dissipated. Secondly, the NES induces energy transfer from the primary structure’s low-frequency modes to the high-frequency regions. Since these frequencies are characterized by higher damping, the excess vibration energy is dissipated by the internal damping of the structure. Employing the methodology described in [27] on the examined drivetrain, numerical evidence for the second mechanism is initially presented. The model represented by Eq. (6) is re-arranged as:

19a $$\begin{aligned}&\mathbf{J}\left[ {{\begin{array}{c} {\ddot{\theta }_2 } \\ {\ddot{\theta }_4 } \\ {\ddot{\theta }_6 } \\ {\ddot{\theta }_7 } \\ {\ddot{\theta }_8 } \\ {\ddot{\theta }_l } \\ {\ddot{\theta }_r } \\ \end{array} }} \right] +\mathbf{C}\left[ {{\begin{array}{c} {\dot{\theta }_2 } \\ {\dot{\theta }_4 } \\ {\dot{\theta }_6 } \\ {\dot{\theta }_7 } \\ {\dot{\theta }_8 } \\ {\dot{\theta }_l } \\ {\dot{\theta }_r } \\ \end{array} }} \right] +\mathbf{K}\left[ {{\begin{array}{c} {\theta _2 } \\ {\theta _4 } \\ {\theta _6 } \\ {\theta _7 } \\ {\theta _8 } \\ {\theta _l } \\ {\theta _r } \\ \end{array} }} \right] \nonumber \\&\qquad +\left[ {{\begin{array}{c} {k_{n2} \left( {\theta _2 -\theta _{n2} } \right) ^{3}+k_{n1} \left( {\theta _2 -\theta _{n1} } \right) ^{3}} \\ 0 \\ 0 \\ 0 \\ 0 \\ 0 \\ 0 \\ \end{array} }} \right] \nonumber \\&\quad =\left[ {{\begin{array}{c} {k_1 \theta _1 +c_1 \dot{\theta }_1 } \\ 0 \\ 0 \\ 0 \\ 0 \\ {\tau _l } \\ {\tau _r } \\ \end{array} }} \right] \end{aligned}$$

19b $$\begin{aligned}&\left[ {{\begin{array}{cc} {J_{n1} }&{} 0 \\ 0&{} {J_{n2} } \\ \end{array} }} \right] \left[ {{\begin{array}{l} {\ddot{\theta }_{n1} } \\ {\ddot{\theta }_{n2} } \\ \end{array} }} \right] +\left[ {{\begin{array}{l} {c_{n1} \left( {\dot{\theta }_{n1} -\dot{\theta }_2 } \right) } \\ {c_{n2} \left( {\dot{\theta }_{n2} -\dot{\theta }_2 } \right) } \\ \end{array} }} \right] \nonumber \\&\quad +\left[ {{\begin{array}{l} {k_{n1} \left( {\theta _{n1} -\theta _2 } \right) ^{3}} \\ {k_{n2} \left( {\theta _{n2} -\theta _2 } \right) ^{3}} \\ \end{array} }} \right] \nonumber \\&\quad =\left[ {{\begin{array}{c} 0 \\ 0 \\ \end{array} }} \right] \end{aligned}$$

Equation (19a) describes the dynamics of the drivetrain, whereas Eq. (19b) represents the dynamics of the two NESs. These equations are coupled through the force produced by the two nonlinear springs $$k_{n1} $$ and $$k_{n2} $$ and the two linear dampers $$c_{n1} $$ and $$c_{n2} $$. Equation (19a) is then changed into modal coordinates using the following transformation:

20 $$\begin{aligned} \left[ {{\begin{array}{c} {\theta _2 } \\ {\theta _4 } \\ {\theta _6 } \\ {\theta _7 } \\ {\theta _8 } \\ {\theta _l } \\ {\theta _r } \\ \end{array} }} \right] =\left[ \mathbf{J} \right] ^{-1/2}\left[ \mathbf{V} \right] \mathbf{q} \end{aligned}$$

where **q** is the 7*x*1 vector of modal coordinates and **V** is the 7*x*7 mass normalized modal matrix. Substituting Eq. (19b) into (19a) and pre-multiplying both sides of the resultant equation by $\left[ \mathbf{V} \right] ^{T}\left[ \mathbf{J} \right] ^{-1/2}$ yields:

21 $$\begin{aligned}&{\ddot{\mathbf{q}}}+\left[ {{\hat{\mathbf{C}}}} \right] {\dot{\mathbf{q}}}+\left[ {{\hat{\mathbf{K}}}} \right] \mathbf{q} =\left[ \mathbf{V} \right] ^{T}\left[ \mathbf{J} \right] ^{-1/2}\left\{ {\left[ {{\begin{array}{c} {k_1 \theta _1 +c_1 \dot{\theta }_1 } \\ 0 \\ 0 \\ 0 \\ 0 \\ {\tau _l } \\ {\tau _r } \\ \end{array} }} \right] }\right. \nonumber \\&\left. \quad -\left[ {{\begin{array}{c} {k_{n2} \left( {\theta _2 -\theta _{n2} } \right) ^{3}+k_{n1} \left( {\theta _2 -\theta _{n1} } \right) ^{3}} \\ 0 \\ 0 \\ 0 \\ 0 \\ 0 \\ 0 \\ \end{array} }} \right] \right\} \end{aligned}$$

where ${\hat{\mathbf{C}}}=\mathbf{V}^{T}{} \mathbf{J}^{-1/2}{} \mathbf{CJ}^{-1/2}\mathbf{V}$ and ${\hat{\mathbf{K}}}=\mathbf{V}^{T}{} \mathbf{J}^{-1/2}\mathbf{KJ}^{-1/2}{} \mathbf{V}$ are 7x7 diagonal matrices. Thus, the instantaneous total mechanical energy for the $q_i$ mode; $E_i$ is computed as:

22 $$\begin{aligned} \left[ {{\begin{array}{c} {E_1 } \\ \vdots \\ {E_7 } \\ \end{array} }} \right] =\frac{1}{2}{\dot{\mathbf{q}}}^{2}+\frac{1}{2}\mathbf{q}^{T}\left[ {{\hat{\mathbf{K}}}} \right] \mathbf{q} \end{aligned}$$

**Fig. 11:**
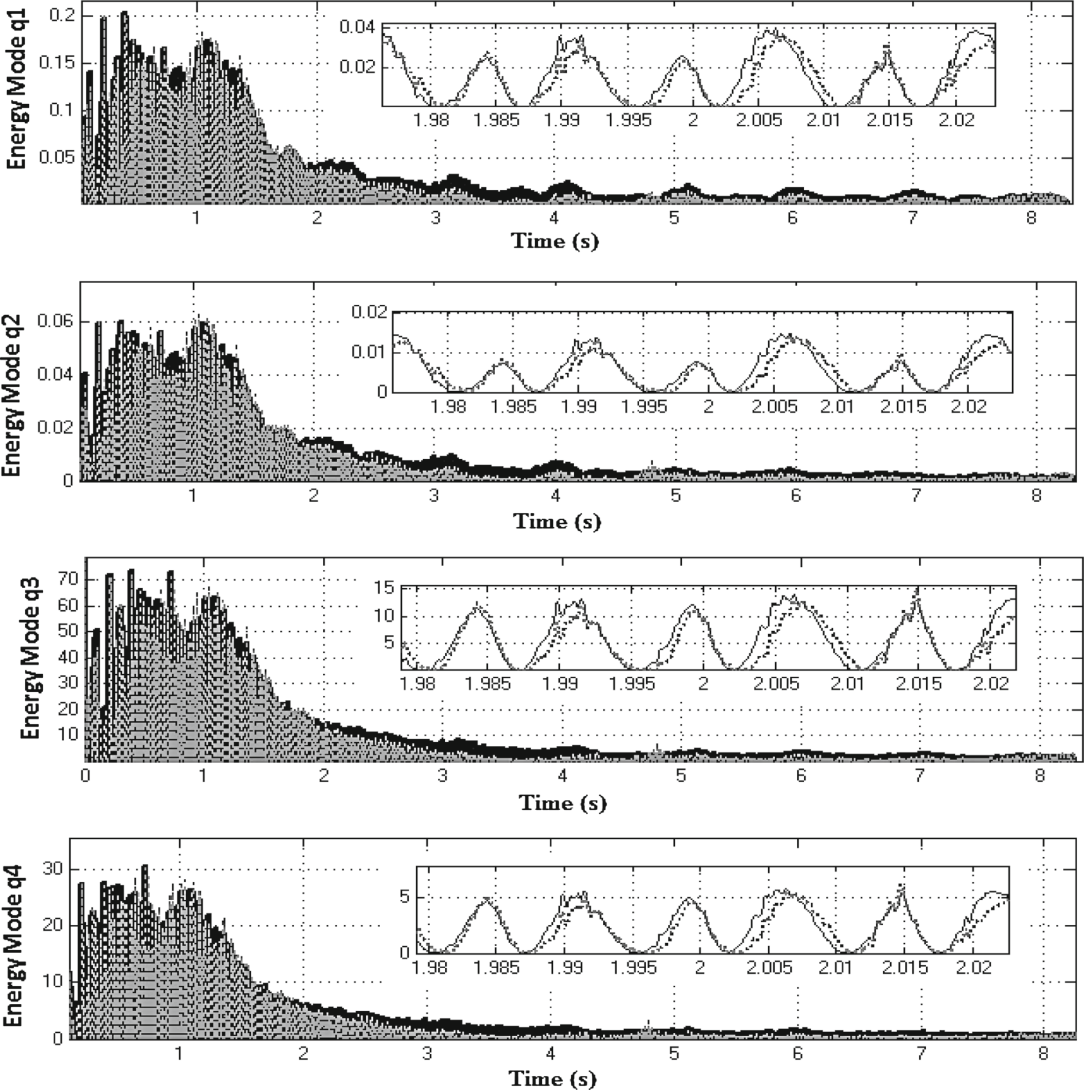
Mechanical energy of the first four modal coordinates for systems with *active* (*dashed line*) and *locked* (*solid line*) NES in first gear with 25 % open throttle manoeuvre (the rigid body modes have been supressed)

**Fig. 12:**
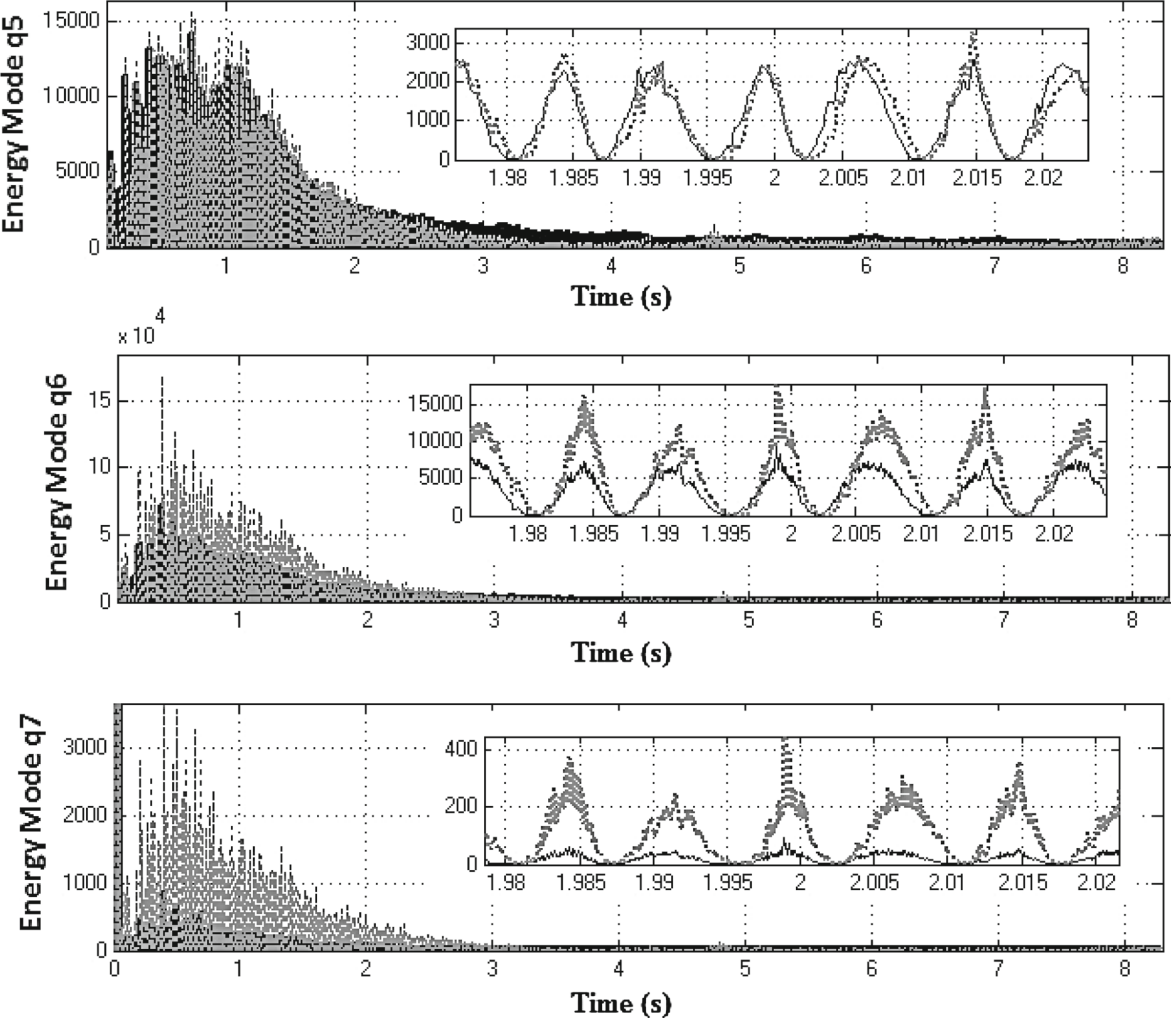
Mechanical energy of modal coordinates $q_{5}, q_{6}$ and $q_{7}$ for systems with *active* (*dashed line*) and *locked* (*solid line*) NES in first gear with 25 % open throttle manoeuvre (the rigid body modes have been supressed)

Energy redistribution between the drivetrain modes can be observed, when the modal energy content is compared for two systems; the *active* and the *locked NES* models (both represented in modal coordinates for this purpose). Both systems are excited using the vehicle manoeuvre already presented in first gear with 25 % open throttle. The optimized set of NES parameters (Table 2) is employed for this exercise. To facilitate the analysis, the rigid body mode for each DoF is suppressed by subtracting the rotation due to the corresponding mean velocity. The latter is computed using the flywheel mean velocity and the corresponding reduction ratio (for the transmission and the differential), where necessary. The first four lower modal coordinates $q_{1}, q_{2},q_{3}$ and $q_{4}$ (where the rigid body mode has been supressed) are used to calculate the corresponding instantaneous mechanical energy of the drivetrain (Fig. 11) for the *locked* system (solid black line) and for the *active* system (dotted grey line). For these modes and within the frequency region, where the two NES are effective (2–8 s of the manoeuvre), it can be seen that the mechanical energy of the *locked* system is slightly higher than that of the *active* system. Snapshots during 2 s time intervals reveal this small difference. Conversely, Fig. 12 shows that the mechanical energy of the three higher-frequency modal coordinates (with suppressed rigid body modes) for *active* NES (dotted grey line) is slightly higher for $q_{5}$ and significantly higher for $q_{6}$ and $q_{7}$, when compared with that of the *locked* system (solid black line). This behaviour clearly demonstrates that the nonlinear vibration absorbers induce energy redistribution between the drivetrain modes (i.e. transferring energy from the lower to higher-frequency modes).

The modal energy redistribution is further extended when calculating the normalized effective damping ratio ${\lambda _{\mathrm{eff},i} }/{\lambda _i }$ [35], where $\lambda _i$ is the modal damping of the system with the *locked* NES. The time-varying effective damping measure [22] becomes:

23 $$\begin{aligned} \lambda _{\mathrm{eff},i} =\frac{\frac{\hbox {d}}{\hbox {d}t}\left( {\left\langle {\dot{q}_i^2 } \right\rangle } \right) }{\left\langle {\dot{q}_i^2 } \right\rangle } \end{aligned}$$

**Fig. 13:**
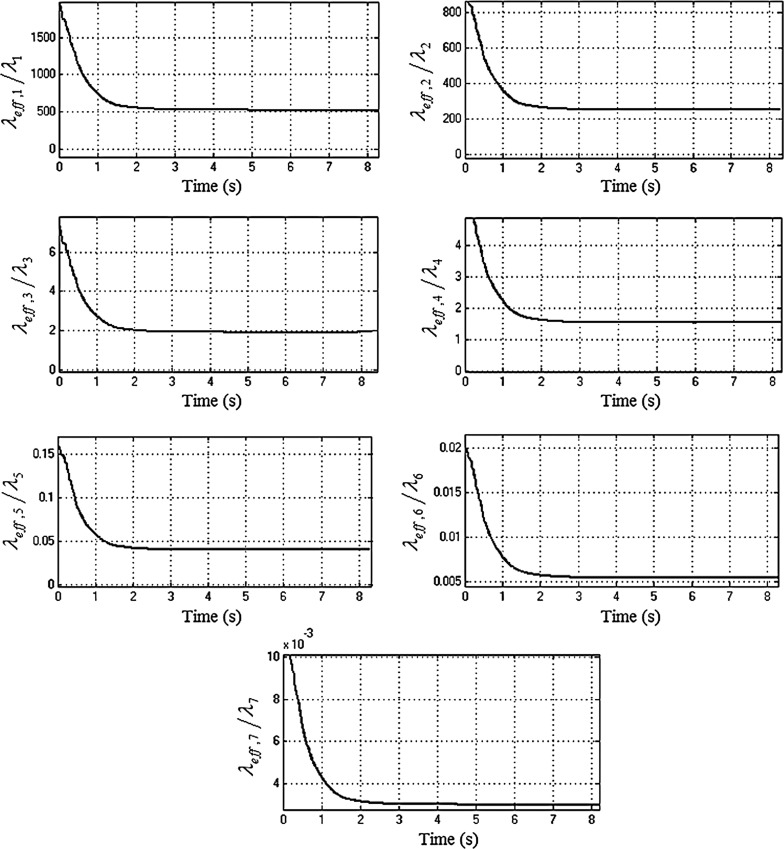
Normalized effective damping of the drivetrain modal coordinates (first gear with 25 % open throttle)

The operator $\left\langle {\dot{q}_i^2 } \right\rangle$ is the envelope of $\dot{q}_i^2$, calculated as a spline fit to the local maxima of $\dot{q}_i^2$. According to [27] if for a particular mode the ratio ${\lambda _{\mathrm{eff},i} }/{\lambda _i }>1$, then energy is transferred from this mode to others, whereas if the ratio ${\lambda _{\mathrm{eff},i} }/{\lambda _i }<1$, then energy is imported from the other modes. Figure 13 shows the normalized effective damping for the seven drivetrain modal coordinates. The first four modes satisfy the condition ${\lambda _{\mathrm{eff},i} }/{\lambda _i }>1$, and it can be concluded that energy is transferred from these low-frequency modes to the higher frequencies. The last three modes satisfy the condition ${\lambda _{\mathrm{eff},i} }/{\lambda _i }<1$, indicating that energy is absorbed by them. These results corroborate the finding that nonlinear absorbers redistribute the energy between the drivetrain modes.

**Fig. 14:**
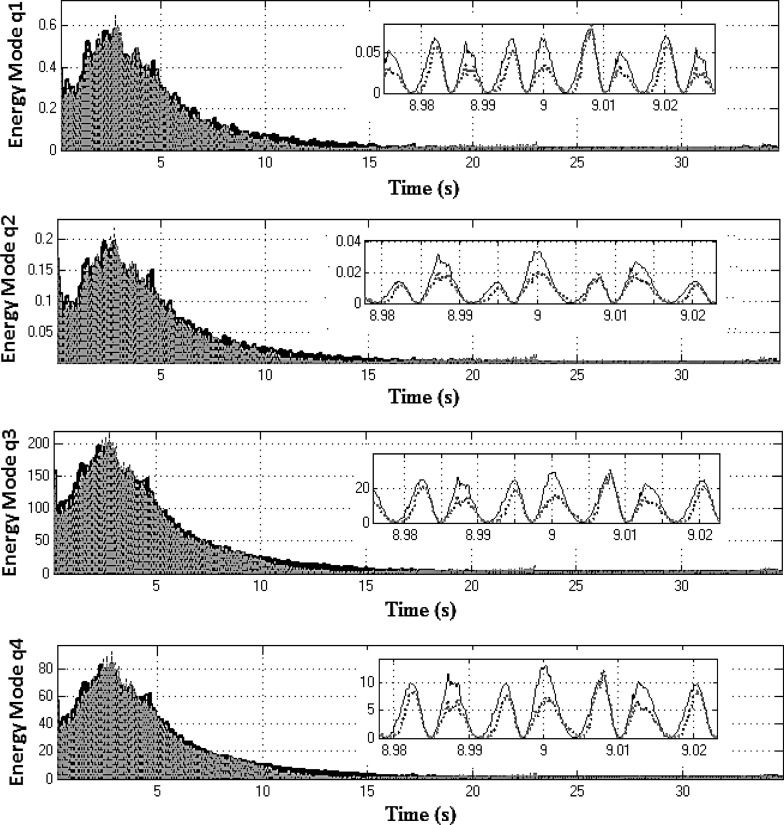
Mechanical energy of the first four modal coordinates for systems with *active* (*dashed line*) and *locked* (*solid line*) NES in third gear with full throttle (the rigid body modes have been supressed)

**Fig. 15:**
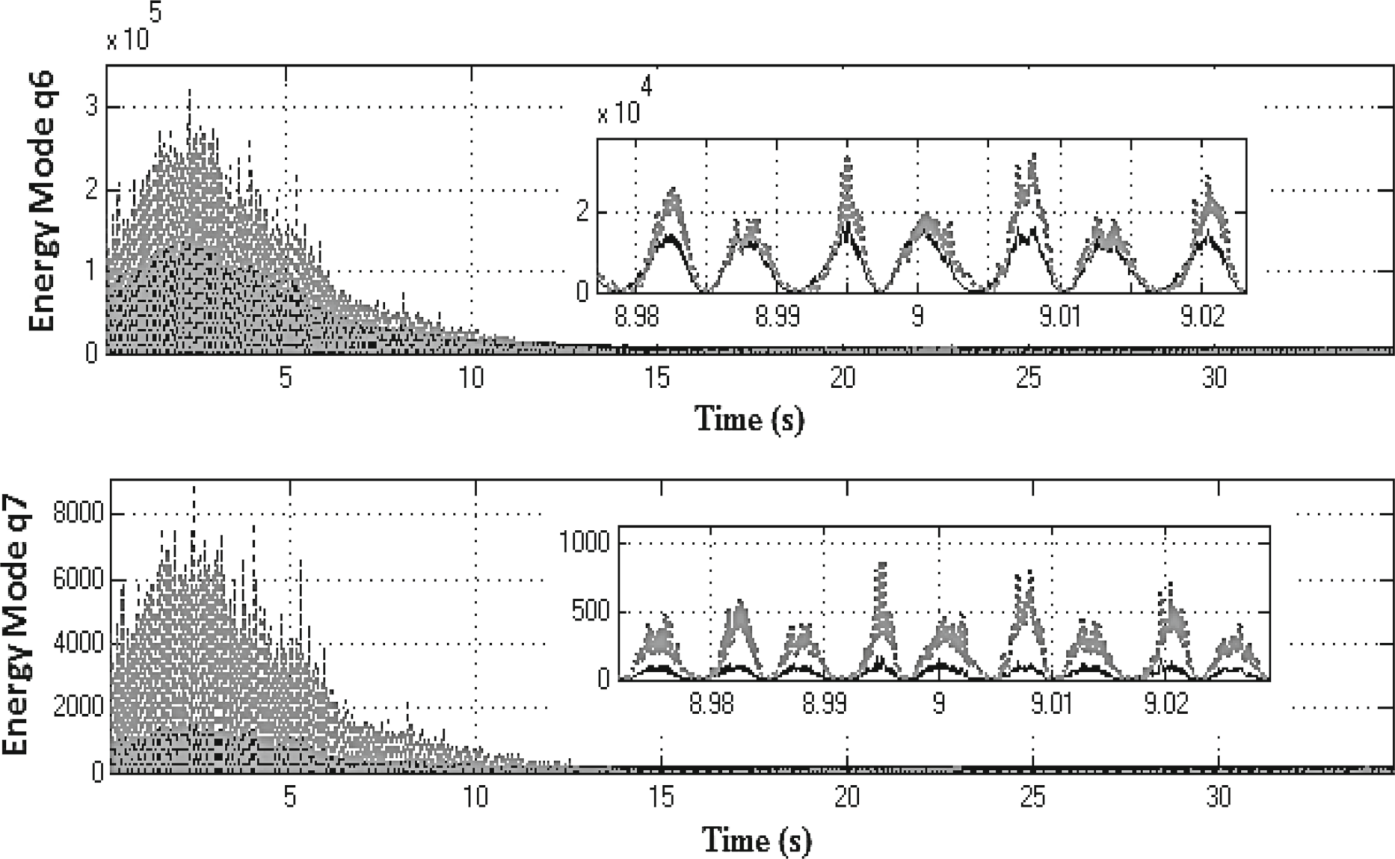
Mechanical energy of the modal coordinates $q_{6}$ and $q_{7}$ for systems with *active* (*dashed line*) and *locked* (*solid line*) NES in third gear with full throttle (the rigid body modes have been supressed)

The previous analysis was repeated for additional manoeuvres engaged in higher gears, and similar results were obtained. The plots in Fig. 14 exhibit the instantaneous mechanical energy of the first four modal coordinates (rigid body modes have been supressed) for *locked* and *active* NES cases for 35 s in third gear at full throttle. For the first four modes, the mechanical energy of the *locked* system is generally higher than that of the *active* system. The insets in the figure during the 9 s time interval clearly show this small difference. Conversely, Fig. 15 shows the mechanical energy of the two higher frequency modal coordinates $q_{6}$ and $q_{7}$ (rigid body mode supressed) of the system with *active* NESs again substantially higher, when compared with those of the *locked* system. This behaviour for the third gear at 100 % open throttle manoeuvre is similar to the previously described manoeuvre in first gear and with 25 % open throttle. Furthermore, the NESs-induced energy redistribution between the drivetrain modes follows the same trend as previously, which is an indication of robust NESs’ operation.

**Fig. 16:**
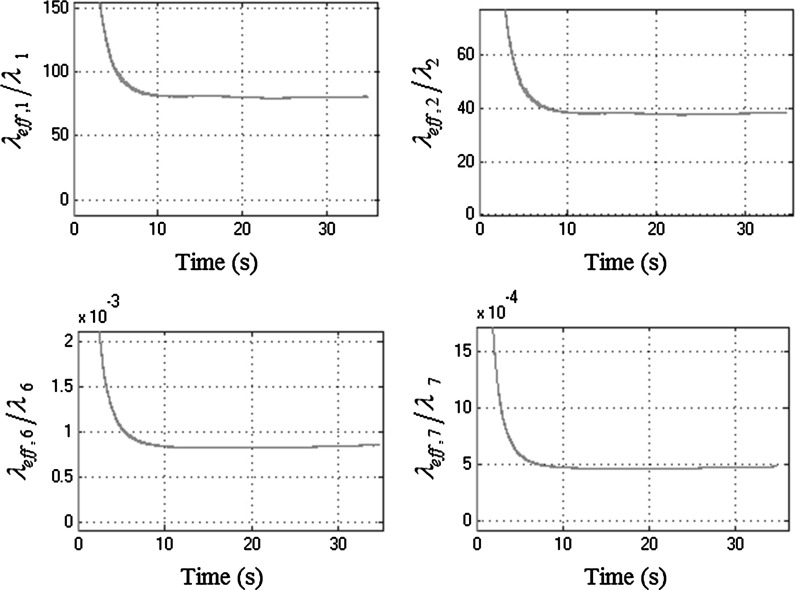
Normalized effective damping of the drivetrain modal coordinates $q_{1}$, $q_{2}$, $q_{6}$ and $q_{7}$ (third gear at full open throttle)

Figure 16 shows the normalized effective damping of four drivetrain modal coordinates for a manoeuvre in third gear engaged with full throttle. Since for the first two modes (i.e. $q_{1}$ and $q_{2}$): ${\lambda _{\mathrm{eff},i} }/{\lambda _i }>1$, then it can be concluded that these modes spread their energy elsewhere throughout the manoeuvre. For the last two modes (i.e. $q_{6}$ and $q_{7}$): ${\lambda _{\mathrm{eff},i} }/{\lambda _i }<1$. Therefore, these modes mainly absorb energy throughout the manoeuvre. Thus, it can be concluded that for this manoeuvre, as in the previous cases, there is energy flow from the lower-frequency modes to the higher ones. These results further verify the effectiveness and consistent action of the nonlinear absorbers in redistribution of energy between the drivetrain modes.

**Fig. 17:**
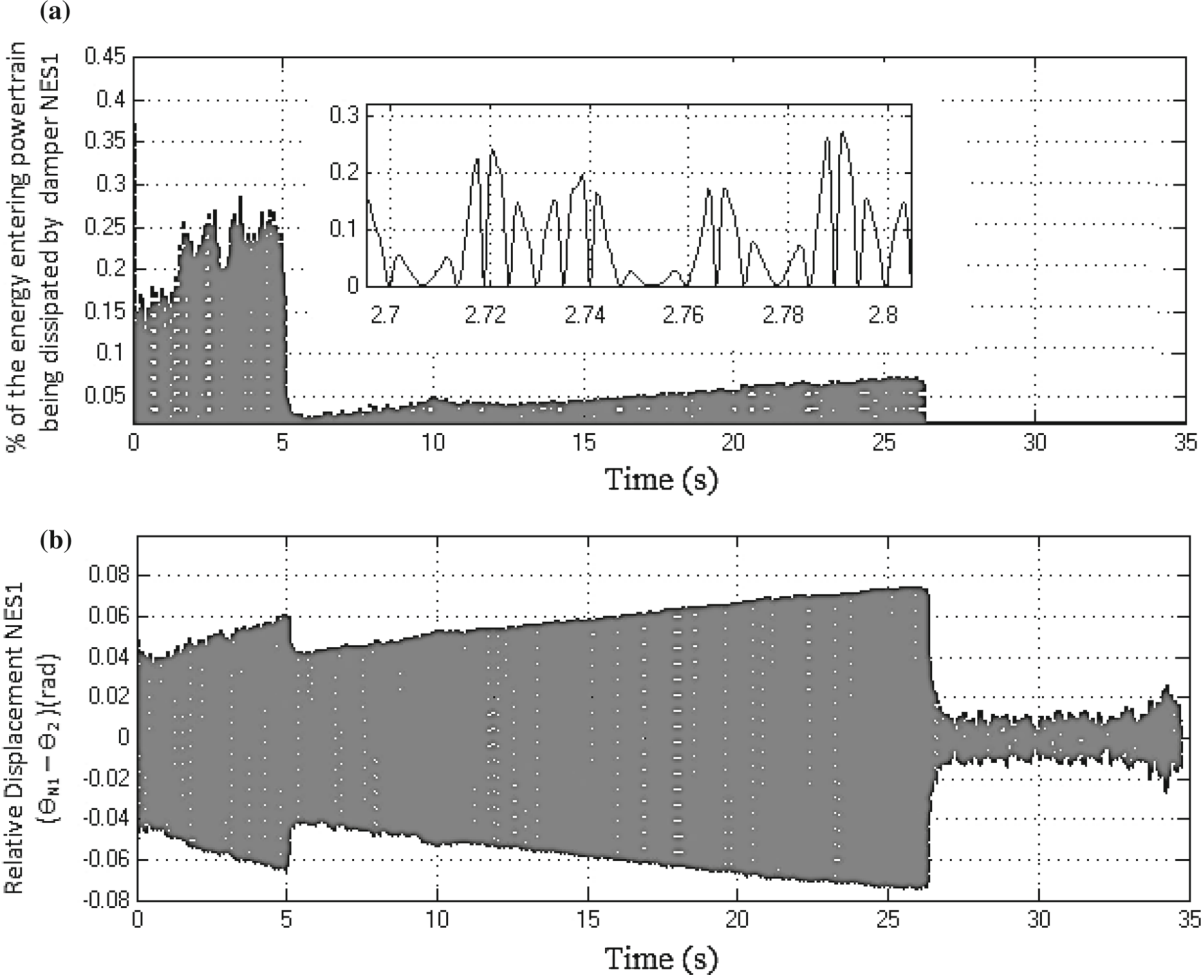
**a** Percentage entrant energy to the drivetrain, dissipated by the NES1 damper. **b** Relative displacement between the NES1 and the clutch (3rd gear full throttle)

**Fig. 18:**
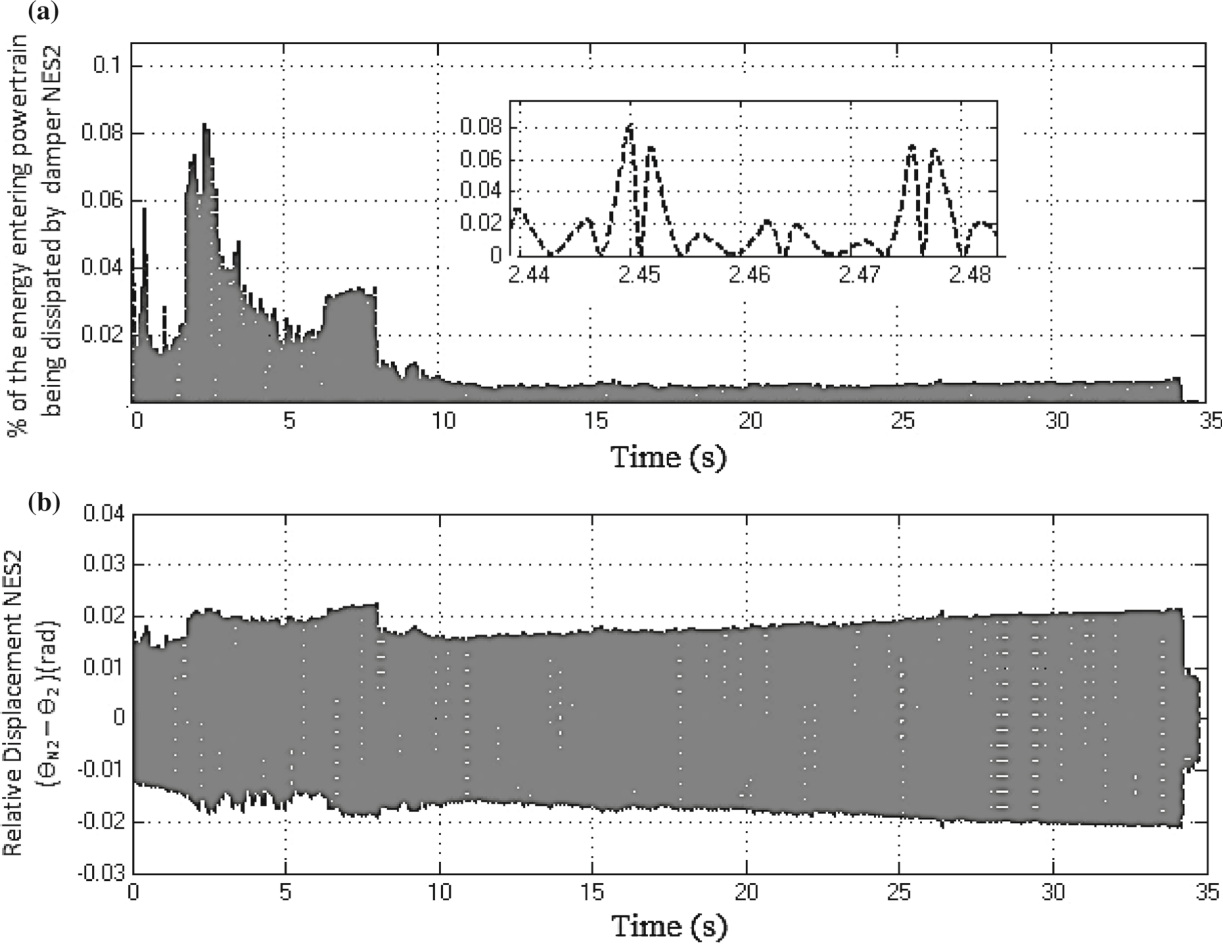
**a** Percentage entrant energy to the drivetrain, dissipated by the NES2 damper. **b** Relative displacement between the NES2 and the clutch (3rd gear at full throttle)

In addition to the modal energy distribution, each NES also induces a single-directional energy transfer from the highly energetic structural modes to the NES, where the energy is then dissipated [42, 43]. Evidence of this mechanism when in third gear at full throttle can be obtained by computing the percentage energy entering into the powertrain which is dissipated by the NES damper(s) [18]. In Eq. (24), the numerator represents the instantaneous dissipated energy by the *i*th damper and the denominator represents the amount of instantaneous entrant energy into the drivetrain.

24 $$\begin{aligned} E_{\mathrm{diss,NES}(i)} \% =100\left( {\frac{c_{\mathrm{ni}} \left( {\dot{\theta }_{n1} -\dot{\theta }_2 } \right) ^{2}}{k_1 \left( {\theta _1 -\theta _2 } \right) ^{2}+J_2 \dot{\theta }_2^2}} \right) \nonumber \\ \end{aligned}$$

Figure 17a shows the percentage entrant energy into the powertrain system which is dissipated by the NES1 damper. In Fig. 17b, NES1 exhibits strong oscillations during the first 25 s of the manoeuvre, but the maximum percentage instantaneous dissipated energy by the absorber in this region is only 0.25 % of the total entrant energy (see the inset to Fig. 17a captured around 2.8 s). Similarly, Fig. 18a shows the percentage entrant energy into the powertrain system, dissipated by the NES2 damper. In this case, NES2 exhibits strong oscillations during the entire manoeuvre, but the dissipated energy is even smaller than in the previous case (only 0.08 % of the total entrant energy, as shown in the inset to Fig. 18a, corresponding to 2.46 s). The above results imply that during the specified manoeuvre most of the excess vibrating energy is dissipated by the structural damping of the drivetrain through modal redistribution.

## Conclusions and future work

The new generations of internal combustion engines induce broadband, high amplitude torsional oscillations, transmitted into the driveline. A novel approach employs the use of nonlinear vibration absorbers coupled to the driveline primary structure. This is in order to reduce the velocity fluctuations by redirecting the excess vibration energy from the lower-frequency drivetrain modes to the higher ones. The paper presents numerical evidence for this energy redistribution process. A validated model of an automotive drivetrain is used to demonstrate the effect of a pair of nonlinear absorbers on the modal energy redistribution of the primary (linear) drivetrain system. The study of the energy content in each modal coordinate shows that in regions where the two parallel NESs reduce the level of vibrations, energy is transferred from lower-frequency to the higher-frequency modes. Since the latter are typically modes with higher damping, the reduction in vibration amplitudes can be directly associated with greater levels of energy dissipation at the higher modes of the structure. This conclusion is further illustrated through estimation of the normalized effective damping coefficients of the different modal coordinates. In particular, the first four lowest drivetrain modes have coefficients exceeding unity, indicating that energy is pumped out of them. Conversely, the higher-frequency modes have normalized effective damping values lower than unity, confirming that energy is absorbed by them. The future direction of this research includes experimental proof of concept, as well as detailed examination of the influence of gear ratio and input load in activation of TET.
